# Cryo-EM structure of human κ-opioid receptor-G_i_ complex bound to an endogenous agonist dynorphin A

**DOI:** 10.1093/procel/pwac033

**Published:** 2022-08-05

**Authors:** Yuxiang Chen, Bo Chen, Tingting Wu, Fangfang Zhou, Fei Xu

**Affiliations:** iHuman Institute, ShanghaiTech University, Shanghai 210210, China; School of Life Science and Technology, ShanghaiTech University, Shanghai 210210, China; Center for Excellence in Molecular Cell Science, Shanghai Institutes for Biological Sciences, Chinese Academy of Sciences, Shanghai 200031, China; University of Chinese Academy of Sciences, Beijing 100049, China; iHuman Institute, ShanghaiTech University, Shanghai 210210, China; iHuman Institute, ShanghaiTech University, Shanghai 210210, China; iHuman Institute, ShanghaiTech University, Shanghai 210210, China; iHuman Institute, ShanghaiTech University, Shanghai 210210, China; School of Life Science and Technology, ShanghaiTech University, Shanghai 210210, China; Center for Excellence in Molecular Cell Science, Shanghai Institutes for Biological Sciences, Chinese Academy of Sciences, Shanghai 200031, China; University of Chinese Academy of Sciences, Beijing 100049, China

**Dear Editor,**

The opioid receptor family is divided into four subtypes each paired with their cognate peptide ligand: the μ-opioid receptor (MOR) with β-endorphin, κ-opioid receptor (KOR) with dynorphin A and B, δ-opioid receptors (DOR) with enkephalin, and nociceptin opioid receptor (NOR) with nociceptin (Faouzi et al., 2020). These four opioid receptors all primarily couple to heterotrimeric G_i_/G_o_ proteins as well as mediating β-arrestin1/2 signaling pathway (Ferre et al., 2019). Activation of these receptors by distinct ligands is linked to a series of physiological responses, such as release of hormones, pain adjustment, drug addiction, stress, and mood regulation (Che et al., 2018).

KOR is widely distributed in the central and peripheral nervous systems. It shares ~60% sequence homology with other opioid receptors, while the sequence differences are mainly located in the extracellular loops, N-terminus and C-terminus (Waldhoer et al., 2004). Activation of KOR by dynorphin and dynorphin-related peptides regulates many physiological actions, including addiction, emotion, and perception (Bruchas et al., 2010).

Dynorphin-A (1–13) is an endogenous peptide derived from the precursor prodynorphin (Ferre et al., 2019). As an enkephalin-like peptide agonist, dynorphin was suggested to have distinct binding mode than the small molecule agonist based on previous mutagenesis and functional studies (Vardy et al., 2013). Targeting the dynorphin/KOR system will hold promise for develop anti-depressant, analgesic, anti-addiction, and anti-anxiety drugs (Ferre et al., 2019). Thus, understanding the molecular recognition for the dynorphin/KOR pair as well as the signaling mechanism of KOR through its cognate downstream G_i_ protein will provide structural basis for developing new-generation peptide-derived ligands and drugs. To facilitate a better understanding of KOR function and activation and to compare with other opioid receptors, we report cryo-EM structure of KOR bound to dynorphin in complex with the G_i_ heterotrimer protein at 3.3 Å resolution (Fig. 1A and 1B; Table S1).

**Figure 1.**
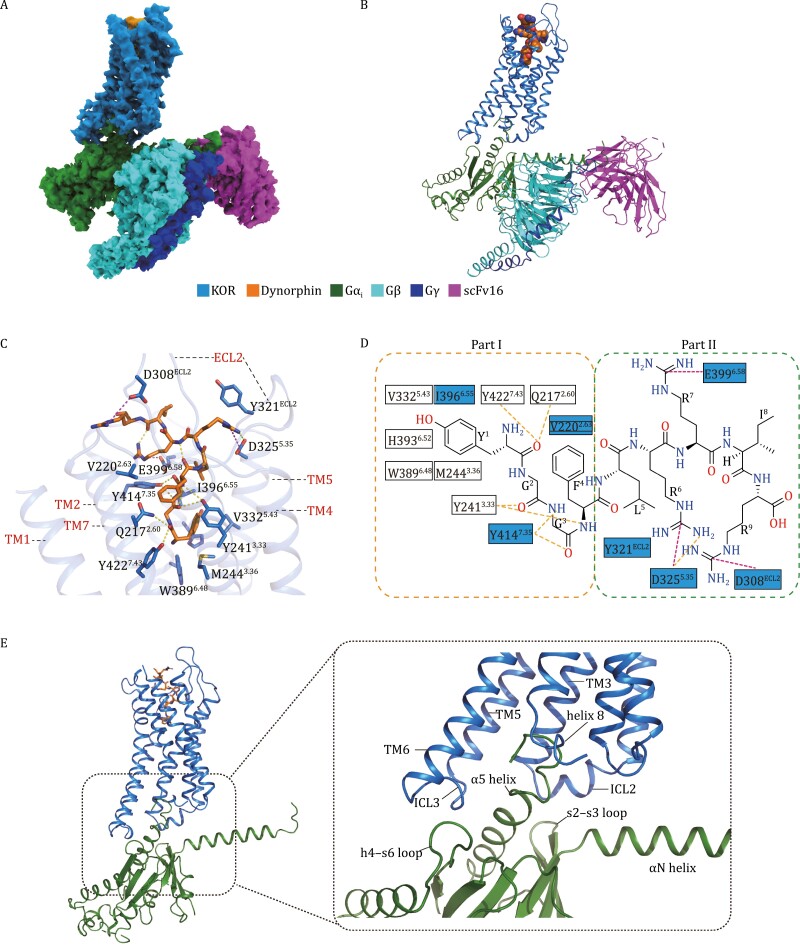
Cryo-EM structure of the KOR-G_i_ complex. (A) The cryo-EM density map of KOR-G_i_ heterotrimer-scFv16 complex colored by subunit (KOR in blue, Gα_i_ in green, Gβ in cyan, Gγ in blue, scFv16 in magenta, and dynorphin in orange). (B) The KOR-G_i_ heterotrimer-scFv16 complex model shown with corresponding color as in (A). (C) Overall binding pocket with dynorphin shown by orange sticks and the interacting residues on KOR in blue sticks. The cartoon was shown as semitransparent to allow clear identification of the peptide and key interactions. Hydrogen bonds are shown as yellow-dashed lines. Magenta-dashed lines represent charge interactions. (D) 2D diagram of molecular interactions between dynorphin and KOR. Residues that vary among the KOR, MOR, and DOR subtypes are highlighted in blue. Dashed lines follow the same coloring rule as in (C). (E) KOR-Gα_i_ interaction interface. KOR is shown in blue and Gα_i_ is in green.

To obtain the dynorphin-bound KOR-G_i_ complex amenable for cryo-EM study, we assembled the purified human KOR protein (residues 54–358) with Gα_i1_, Gβ_1_γ_2_, and scFv16 proteins in the presence of dynorphin-A (1–13) (Fig. S1). The cryo-EM map is finally refined to 3.3 Å resolution allowing accurate assignment of each protein component and the peptide ligand into the corresponding densities (Figs. 1A, 1B, S2 and S3).

In contrast to the binding orientation of apelin in APJR where the C-terminal tail inserts into the transmembrane (TM) core (Ma et al., 2017), dynorphin uses the N-terminal part to relay the signaling and engage the major molecular interactions with KOR while the C-terminal part extends to the extracellular surface. While the first nine residues of dynorphin-A containing Tyr1 to Arg9 can be unambitiously fit into the density map, the C-terminal tail (R10–R13) is not modeled likely owing to its flexibility. Three intramolecular hydrogen bonds stabilized the dynorphin in an S shape similar to the previously reported peptides in bradykinin B1R and B2R structures (Yin et al., 2021) (Fig. 1C and 1D). The primary interface between KOR and G_i_ heterotrimer is composed of ICL2, ICL3, TMs 3, 5, 6 and C-terminal helix 8 on the receptor and the α5 helix, s2–s3 loop, h4–s6 loop on the Gα subunit of the G_i_ protein (Fig. 1E). The overall organization of KOR-G_i_ is similar to the MOR-G_i_ structure except for a ~5° rotation of Gα subunit between the two complexes (Fig. S4). Residues on ICL2 and ICL3 of KOR involved in the KOR and G_i_ interface are conserved among opioid receptors (Fig. S5), suggesting a common binding mode between opioid receptors and Gα_i_ protein.

The primary interaction residues around the binding cavity of dynorphin are located on TMs 2, 3, 5, 6, 7 and extracellular region of KOR (Fig. 1C). We divided these interactions into two parts based on the sequence properties of dynorphin (Fig. 1D). The “part I” interactions are formed by hydrophobic, hydrogen bonding between dynorphin “Y^1^G^2^G^3^F^4^” residues and KOR. The “part II” interactions are primarily charge interactions composed of the “L^5^R^6^R^7^I^8^R^9^” residues in dynorphin and negatively-charged residues in KOR on the extracellular region.

In “part I” interactions, dynorphin N-terminal Tyr1 extends its phenol group into the hydrophobic space at the bottom of the cavity. Previous studies confirmed that truncation of the N-terminal residue Tyr1 of dynorphin abolished its biologic activity (Chavkin and Goldstein 1981) (Fig. 1C and 1D). Our mutagenesis and functional analysis confirmed that mutations of these conserved residues (W389^6.48^, M244^3.36^, V322^5.43^, H393^6.52^, and Y241^3.33^) of KOR decreased the potency of dynorphin in the cAMP functional assay (Table S2). Another important interaction contributing to the hydrogen bonds network is formed by Y241^3.33^ of KOR with dynorphin Gly2 and Phe4 main chain groups, Y414^7.35^ with Gly3 main chain group, as well as Q217^2.60^ and Y422^7.43^ with Tyr1 main chain group (Fig. 1C and 1D). Mutations of the residues engaged in the hydrogen bonds network impaired the dynorphin induced G_i_ signaling activity (Table S2). Previous studies also showed that single mutations of Q217^2.60^A, Y414^7.35^A, and Y422^7.43^A led to declined binding affinity of dynorphin to KOR (Ferre et al., 2019).

In “part II” interactions, the positively charged residues Arg6, Arg7, and Arg9 of dynorphin form extensive polar interactions with KOR (Fig. 1C and 1D). For example, Arg6 has charge interaction with D325^5.35^ and also makes a cation–π interaction with Y321^ECL2^ on the KOR extracellular loop 2 (ECL2). Similarly, Arg7 has a charge interaction with E399^6.58^ and also forms a cation–π interaction with Y414^7.35^. Arg9 has a charge interaction with ECL2 residue D308^ECL2^. Mutagenesis and functional experiments confirmed that mutations of these “part II” interacting residues in KOR led to impaired G_i_ signaling in response to dynorphin (Table S2). Notably, previous studies proposed that the sequence divergence on the ECL2 among opioid receptors may encode the key determinants for different affinities of dynorphin-receptor pairs (Ferre et al., 2019). Our structural observations of the key interactions between dynorphin and ECL2 of KOR, further confirmed these hypotheses. This finding also highlights the importance of the “part II” sequence on the peptide for opioid receptor recognition and may provide insights for novel peptide ligand design.

Next, we compared the dynorphin-bound G_i_-coupled KOR structure with previously reported active-state MP1104-KOR-nanobody complex structure (Che et al., 2018) (Fig. S6). The cytoplasmic end of TM6 along with ICL3 in dynorphin-KOR structure displays a further outward movement by ~3 Å compared with that in the MP1104-KOR structure, which is a result of accommodating the Gα_i_ protein. At the extracellular surface, TM6 shows an outward movement by about 2.5 Å to accommodate the peptide binding. In addition, peptide binding induced movements were observed at the extracellular region including shifts on ECL1, ECL2 and ECL3 by 3, 5, and 7 Å, respectively. These ECL movements are consistent with the extracellular interactions between KOR and dynorphin.

To gain further insight into the peptide binding mode in the opioid systems, we compared the peptide binding cavities among three opioid receptors. The overall structure of dynorphin activated KOR adopts an active conformation consistent with DAMGO-bound MOR and KGCHM07-bound DOR structures (Koehl et al., 2018; Claff et al., 2019) (Fig. 2A). In accordance with that, all three opioid receptors display a conserved pocket for accommodating dynorphin (in KOR), DAMGO (in MOR), and KGCHM07 (in DOR), which is primarily composed of residues on TM2, 3, 5, 6, and 7, and partially covered by the extracellular loops, suggesting a common orthosteric binding site in different opioid receptors.

**Figure 2.**
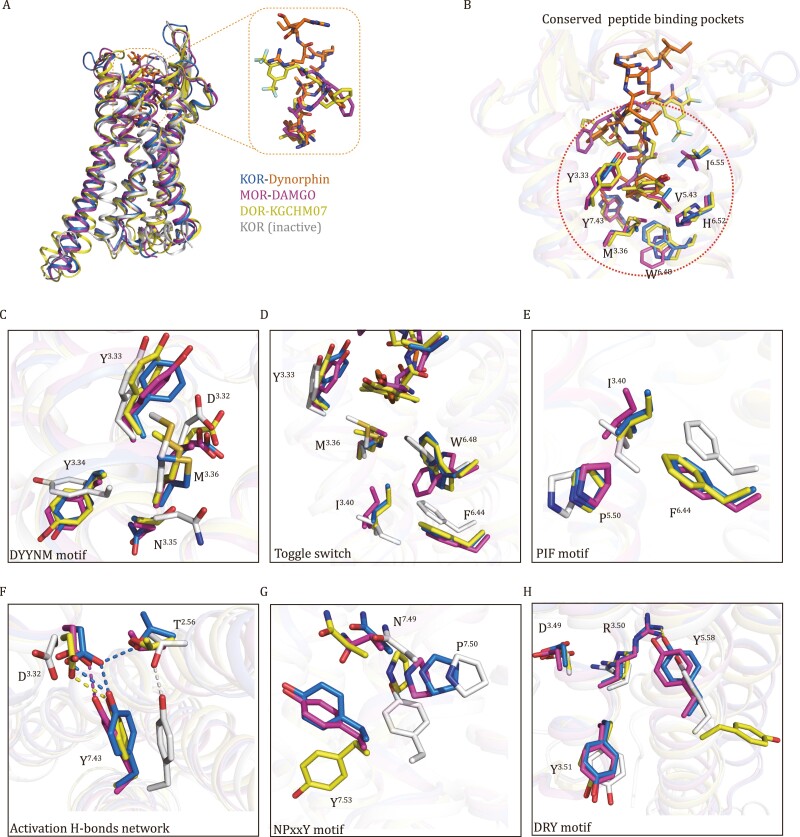
Structural comparison among dynorphin-bound KOR, inactive-state KOR, DAMGO-bound MOR and KGCHM07-bound DOR. (A) Overall structural alignment of dynorphin-KOR (orange-blue), inactive-state KOR (grey, PDB: 4DJH), DAMGO-MOR (magenta-magenta, active-state, PDB: 6DDE) and KGCHM07-DOR (yellow-yellow, active-state, PDB: 6PT2). (B) Conserved peptide binding pockets in different opioid receptors. (C), (D), (E), (F), (G), and (H) Rearrangements of key residues and motifs during dynorphin induced KOR activation and comparison to other opioid receptors. Blue, dynorphin-bound active-state KOR; grey, inactive-state KOR; magenta, active-state MOR; yellow, active-state DOR.

One remarkable feature for the three peptides is that they all place the common Tyr1 residue at the identical position in the binding pockets with the same depth, suggesting the governing role of this observed interaction in our dynorphin-KOR-G_i_ structure. Moreover, the interacting residues around Tyr1 on the peptides are conserved in all three opioid receptors (Fig. S5), allowing them to stabilize the peptide in a similar pose (Fig. 2B). Compared to the two shorter peptide derived agonists, dynorphin extends its “part II” interactions, through Leu^5^-Arg^9^, toward the extracellular surface with highly divergent sequence at extracellular loops and TM bundle residues. As a result, the binding interface for dynorphin is the largest among the three peptide activated opioid receptor complexes (Fig. 2B).

We also observed common peptide activation mechanism of opioid receptors by structural comparison. For the opioid system, peptide entry into the binding pocket is through its “head” benzene right that makes extensive interactions with Y^3.33^, M^3.36^ (D^3.32^Y^3.33^xxM^3.36^ motif) and the toggle switch W^6.48^, causing rearrangements of these side chains (Fig. 2B–D). The rearrangement of W^6.48^, through making new interaction with F^6.44^, triggers the outward movement of the cytoplasmic end of TM6. In addition, P^5.50^ moves inward, I^3.40^ changes its side chain rotameric state, and F^6.44^ moves outward (Fig. 2E). We noticed another activation-related H-bonds interaction network formed by residues Y^7.43^, D^3.32^, and T^2.56^ (Fig. 2F). In the dynorphin-KOR complex structure, Y422^7.43^ moves by 3.2 Å when compared with the JDTic-bound KOR structure, thus breaking the hydrogen bond with T213^2.56^ present in the inactive state. This movement initiates the inward shift of TM7, followed by rearrangement of several residues on the TM7, including N^7.49^, P^7.50^, Y^7.53^ on the activation-conserved NPxxY motif (Fig. 2G). Our mutagenesis and functional studies showed that Y422^7.43^A mutation decreased the potency of dynorphin in the cAMP signaling by more than 50-fold (Table S2), suggesting an integral role of this residue in KOR activation. Indeed, the extra isopropyl group in the antagonist JDTic would generate strong clash with residue Y422^7.43^ when placed into the active-state conformation (Fig. S7). This phenomenon may explain the mechanism of opposite functional properties between MP1104 and JDTic when both contain a similar annular hydrophobic core. DRY motif also shows rearrangements at the intracellular end of TM3, allowing R^3.50^ to form a hydrogen bond with Y^5.58^ (Fig. 2H). Together, these conserved movements of activation-related residues and motifs among three opioid receptors provide a common activation mechanism induced by peptide agonists.

KOR has been a drug target ranging from analgesics, depression, and alcohol dependence (Wee and Koob 2010; Chavkin 2011). Many peptide derivatives have been designed to target KOR, none has advanced to clinical efficacy stage, owing to the difficulty of development with the lack of an accurate structural template. Here we provide the first structural model for dynorphin activated KOR. Such a model shows both common and distinct mechanism with other opioid receptors, providing a framework for developing selective and potent peptide ligands.

Furthermore, our structure reveals a novel “two-part” binding mode for dynorphin, which was previously proposed by NMR, biophysical, and computational studies (O’Connor et al., 2015). The N-terminal “message” part-I and C-terminal “address” part-II sequences on dynorphin and the respective interactions with KOR decipher the mechanism of signal relaying from the ligand to the receptor and serve as a scaffold for future peptide design. Previous studies suggest that G protein signaling is beneficial while β-arrestin activation might be undesired to confer the analgesic effect of KOR agonists (Manglik et al., 2016). Therefore, understanding the structural basis of peptide-KOR-G_i_ complex organization is important to guide rational design of G-protein biased agonist or modulator to overcome on-target side effects.

## Supplementary Material

pwac033_suppl_Supplementary_Material

## Footnotes

The atomic coordinates of the KOR-Gi complex have been deposited in the Protein Data Bank with the accession codes 7Y1F. The EM maps for KOR-Gi complex have been deposited in EMDB with codes EMD-33562. All other data are available upon request to the corresponding author.

This work was supported by the Ministry of Science and Technology of China (Grant No. 2018YFA0507000 to F.X.), the National Natural Science Foundation of China (Grant Nos. 32071194, 32111530085, and 81861128023 to F.X.), and the Science and Technology Commission of Shanghai Municipality (Grant No. 19XD1422800 to F.X.). The cryo-EM data were collected at Shuimu BioSciences Ltd and Bio-EM Facility of ShanghaiTech University. We thank Q. Tan, J. Liu, N. Chen, Q. Shi and W. Xiao from iHuman Institute for functional assay, protein cloning and expression support; Q. Sun, Y. Wang, Y. Liu and Z. Zhang at the Bio-EM facility at ShanghaiTech University for technical support on data collection.

Y.C. performed cloning, protein purification, cryo-EM sample preparation, data collection, structure analysis and figure preparation. B.C. performed cryo-EM sample preparation, data collection, data processing, structure analysis, model building, refinement and figure preparation. T.W. performed cloning, protein purification and cryo-EM sample preparation. F.Z. assisted with cell-based functional assays; F.X. conceived the project. F.X. designed, coordinated and supervised the experiments. Y.C., B.C., and F.X. wrote the manuscript.

All the authors declare that they have no conflict of interest. This article does not contain any studies with human or animal subjects.
