# Supplementary material for: Cryo-EM structure of human κ-opioid receptor-Gi complex bound to an endogenous agonist dynorphin A
Source: Protein Cell. 2022 Aug 5;14(6):464–8. doi: 10.1093/procel/pwac033 (PMC10246719; doi:10.1093/procel/pwac033)

**Supplemental Material**

**Materials and methods**

**Molecular cloning of KOR constructs**

The human KOR gene was subcloned into the expression vector pFastBac1 vector with an N-terminal HA-Flag tag, followed by a 10x His tag and a TEV cleavage site. Thermostabilized apocytochrome b_562_RIL (BRIL) was inserted into the N-terminal of KOR to replace the N-terminal residues M1-H53. C-terminal residues 359-380 of KOR were removed, I135^3.29^L mutation was introduced to improve the protein expression. This construct was designed as previously used in the MP1104-KOR structure (Che et al., 2018).

**KOR protein expression and purification**

We used the Bac-to-Bac baculovirus system (Invitrogen) in Spodoptera frugiperda (Sf9) cells for expression. These cells were infected with baculovirus at a density of 2 × 10^6^ cells per ml. 25 mM of the antagonist naltrexone (NTX) was added during expression. Cells were grown at 27 °C and collected 48 h after infection. The cell membrane was washed with a low-salt buffer (10 mM HEPES (pH 7.5), 20 mM KCl, 10 mM MgCl_2_ and protease inhibitor cocktail (Roche)) and two times with a high-salt buffer (10 mM HEPES (pH 7.5), 1 M NaCl, 20 mM KCl, 10 mM MgCl_2_ and protease inhibitor cocktail). The membranes were resuspended with 25 μM NTX and 2 mg/ml iodoacetamide (Sigma) at 4 °C for 30min. The membranes were then solubilized in solubilization buffer ((10 mM HEPES (pH 7.5), 20 mM KCl, 10 mM MgCl_2_, 2% (w/v) lauryl maltose neopentyl glycol (LMNG; Anatrace) and 0.2% (w/v) cholesteryl hemisuccinate (CHS) (Sigma)) at a ratio of 1:1 at 4 °C for 2 h. The sample was centrifuged at 160,000 g for 30 min to remove debris, and the supernatant was incubated with TALON IMAC resin (Clontech) at 4 °C overnight. Then, the resin was washed with 10 column volumes of 50 mM buffer I ((HEPES (pH 7.5), 500 mM NaCl, 10% (v/v) glycerol, 1% (w/v) LMNG, 10 mM MgCl_2_, 10 mM imidazole), followed by 6 column volumes buffer II ((50 mM HEPES (pH 7.5), 500 mM NaCl, 10% (v/v) glycerol, 0.05% (w/v) LMNG and 40 mM imidazole). The receptor was then eluted with 4 column volumes buffer III (25 mM HEPES (pH 7.5), 500 mM NaCl, 10% (v/v) glycerol, 0.01% (w/v) LMNG and 300 mM imidazole). The protein solution was concentrated to a volume of 0.5 ml and loaded onto a Superdex 200 10/300 column (GE Healthcare) equilibrated with buffer IV (20 mM HEPES (pH 7.5), 100 mM NaCl, 0.01% (w/v) LMNG). The monomer peak was collected for assembling the final complex.

**Purification and formation of the KOR-G_i_-scFv16 complex**

Purification of scFv16 and human G_i_ heterotrimer proteins was conducted as previously described (Hua et al., 2020; Koehl et al., 2018). Purified KOR was mixed with a 10-fold molar excess of dynorphin, 2-fold molar excess of heterotrimeric G_i_ heterotrimer proteins and 1.5-fold molar excess of scFv16 in the presence of apyrase (0.2 U/ml). The mixture was incubated on ice overnight. The sample was loaded on to a Superdex 200 10/300 column. Peak fractions containing the KOR–G_i_–scFv16 complex were pooled and concentrated to 1.2 mg/ml.

**Preparation of Cryo-EM sample**

A 3.5 μL droplet of purified sample at a concentration of around 1.2 mg/ml was applied to glow-discharged holy carbon grids (Quantifoil, 300 mesh gold R1.2/1.3). Excess sample was removed by blotting with filter paper for 1.5 s. Then the plunge-freezing in liquid ethane using a FEI Vitrobot Mark IV at 100% humidity and 4 °C.

**Cryo-EM Data Acquisition**

The dataset of KOR-Gi was collected on a FEI Titan Krios G3i microscope (Thermo Fisher Scientifics, USA) equipped with a GIF Quantum energy filter (20 eV energy slit width, Gatan Inc., USA) and a K3 direct electron detector (Gatan Inc, USA). A total of 2983 Movies were recorded (calibrated pixel size: 1.08 Å/pixel) at the super resolution counting mode by EPU. A 70 μm C2 aperture was always inserted during the data collection period. The defocus ranges from -0.7 to -2.0 μm. For each movie stack, a total of 40 frames are recorded at a dose rate of 20 e^-^/pixel/sec for a duration of 3.5 s, yielding a total dose of 60 e^-^/A^2^.

**Data Processing**

The dataset were motion corrected with MotionCor2 (Zheng et al., 2017) using 7 x 5 patches and no frame grouping. Both the dose weighted and non-dose weighted averages were saved, and the CTF parameters were estimated based on the non-dose-weighted averages using CTFFind (Rohou & Grigorieff, 2015). Only images with highest resolution better than 4.5 Å were selected for further processing. Moreover, images with empty holes, visible contamination or large carbon regions by manual examination were also removed. A total of 2877 movies were finally chosen for particle picking. To avoid potential bias about the structural conformation in the dataset, a Laplacian-of-Gaussian blob picker was firstly applied to pick particles. Good 2D class averages with randomized orientations and clear secondary features were selected as the 2D templates for another round of autopicking process, yielding an initial particle stack of 2,919,730 particles. Further round of 2D classification were applied to eliminate bad quality particles by Relion 3.1 (Scheres, 2012), yielding a dataset containing 1,518,871 good quality particles with randomized orientations and clear secondary features. The recently published Fzd7-G_s_ complex cryo-EM map was firstly chosen as the initial template for 3D refinement (Xu et al., 2021). For the first round 3D classification, this initial model was firstly applied a low-pass filter of 40 Å and used to divide the dataset into 3 different 3D classes. Subsequent rounds of 3D classification of good classes with masks generated by EMAN2 (Tang et al., 2007) focusing on the receptor further separated particles containing higher resolution info from ones with lower resolution info. Finally, a total of 893,618 particles were selected for homogeneous refinement, yielding a map of 3.32 Å resolution. Post-processing further improves the resolution to 3.27 Å at a Fourier shell correlation of 0.143. The local resolution was estimated using the cryosparc v2.15 “local resolution estimation” function.

**Model Building**

The homology models of the KOR and G_i_ were initially generated by the Swiss model (Guex, Peitsch, & Schwede, 2009) (template: 6DDE). The Gβ, Gγ and scFv16 structures were also from 6dde, while the dynorphin model was built from the 2n2f. These models were then fitted into the density maps in UCSF Chimera (Pettersen et al., 2004), and manually adjusted to fit the density maps in Coot software (Emsley & Cowtan, 2004). Subsequently, the generated model was automatically refined and manually adjusted in Coot and Phenix (Adams et al., 2010), respectively, for several iterations. The clashscores, Molprobity and Ramachandran analysis was performed using the MolProbity (Williams et al., 2018). The final refinement statistics were generated using the “comprehensive validation (cryo-EM)” function in Phenix. Structural figures were prepared in Chimera, coot and Pymol.

**cAMP functional assay**

HEK-293T cells were seeded into 6-well cell culture plates. After overnight culture, the cells were co-transfected with KOR WT vector (KOR mutation vector) and pGlosenser^TM^-22F cAMP plasmid (Promega) using the calcium phosphate transfection method. After 24 h, the transfected cells were seeded into poly-D-lysine coated 384-well white clear bottom cell culture plates (15,000 cells per well) in DMEM supplemented with 1% dialysed FBS (40 μL per well). Cells were incubated for a further 24 h at 37 °C in a 5% CO_2_ incubator. On the day of assay, growth medium was removed and cells were loaded with 20 μL of luciferin prepared in assay buffer for 60 min at 37 °C. 10 μL 3x drug solutions were added for 15 min before addition of 10 μL isoproterenol at a final concentration of 200 nM. Plates were then incubated for 15 min at room temperature and chemiluminescence were measured by an Envision multilabel plate reader (PerkinElmer). Results were analyzed in GraphPad Prism (v8.0; GraphPad Software Inc.) by nonlinear regression (curve fit) to calculate each logEC_50_ (negative value of pEC_50_). Followed by calculating the mean value of pEC_50_ and standard error of mean (S.E.M.) of several independent experiments (n = 2-7) via MS. Excel.

**References**

Adams, P. D., Afonine, P. V., Bunkoczi, G., Chen, V. B., Davis, I. W., Echols, N., . . . Zwart, P. H. (2010). PHENIX: a comprehensive Python-based system for macromolecular structure solution. *Acta Crystallogr D Biol Crystallogr, 66*(Pt 2), 213-221. doi:10.1107/S0907444909052925

Che, T., Majumdar, S., Zaidi, S. A., Ondachi, P., McCorvy, J. D., Wang, S., . . . Roth, B. L. (2018). Structure of the Nanobody-Stabilized Active State of the Kappa Opioid Receptor. *Cell, 172*(1-2), 55-67 e15. doi:10.1016/j.cell.2017.12.011

Emsley, P., & Cowtan, K. (2004). Coot: model-building tools for molecular graphics. *Acta Crystallogr D Biol Crystallogr, 60*(Pt 12 Pt 1), 2126-2132. doi:10.1107/S0907444904019158

Guex, N., Peitsch, M. C., & Schwede, T. (2009). Automated comparative protein structure modeling with SWISS-MODEL and Swiss-PdbViewer: a historical perspective. *Electrophoresis, 30 Suppl 1*, S162-173. doi:10.1002/elps.200900140

Hua, T., Li, X., Wu, L., Iliopoulos-Tsoutsouvas, C., Wang, Y., Wu, M., . . . Liu, Z. J. (2020). Activation and Signaling Mechanism Revealed by Cannabinoid Receptor-Gi Complex Structures. *Cell, 180*(4), 655-665 e618. doi:10.1016/j.cell.2020.01.008

Koehl, A., Hu, H., Maeda, S., Zhang, Y., Qu, Q., Paggi, J. M., . . . Kobilka, B. K. (2018). Structure of the micro-opioid receptor-Gi protein complex. *Nature, 558*(7711), 547-552. doi:10.1038/s41586-018-0219-7

Pettersen, E. F., Goddard, T. D., Huang, C. C., Couch, G. S., Greenblatt, D. M., Meng, E. C., & Ferrin, T. E. (2004). UCSF Chimera--a visualization system for exploratory research and analysis. *J Comput Chem, 25*(13), 1605-1612. doi:10.1002/jcc.20084

Rohou, A., & Grigorieff, N. (2015). CTFFIND4: Fast and accurate defocus estimation from electron micrographs. *J Struct Biol, 192*(2), 216-221. doi:10.1016/j.jsb.2015.08.008

Scheres, S. H. (2012). RELION: implementation of a Bayesian approach to cryo-EM structure determination. *J Struct Biol, 180*(3), 519-530. doi:10.1016/j.jsb.2012.09.006

Tang, G., Peng, L., Baldwin, P. R., Mann, D. S., Jiang, W., Rees, I., & Ludtke, S. J. (2007). EMAN2: an extensible image processing suite for electron microscopy. *J Struct Biol, 157*(1), 38-46. doi:10.1016/j.jsb.2006.05.009

Williams, C. J., Headd, J. J., Moriarty, N. W., Prisant, M. G., Videau, L. L., Deis, L. N., . . . Richardson, D. C. (2018). MolProbity: More and better reference data for improved all-atom structure validation. *Protein Sci, 27*(1), 293-315. doi:10.1002/pro.3330

Xu, L., Chen, B., Schihada, H., Wright, S. C., Turku, A., Wu, Y., . . . Xu, F. (2021). Cryo-EM structure of constitutively active human Frizzled 7 in complex with heterotrimeric Gs. *Cell Res*. doi:10.1038/s41422-021-00525-6

Zheng, S. Q., Palovcak, E., Armache, J. P., Verba, K. A., Cheng, Y., & Agard, D. A. (2017). MotionCor2: anisotropic correction of beam-induced motion for improved cryo-electron microscopy. *Nat Methods, 14*(4), 331-332. doi:10.1038/nmeth.4193


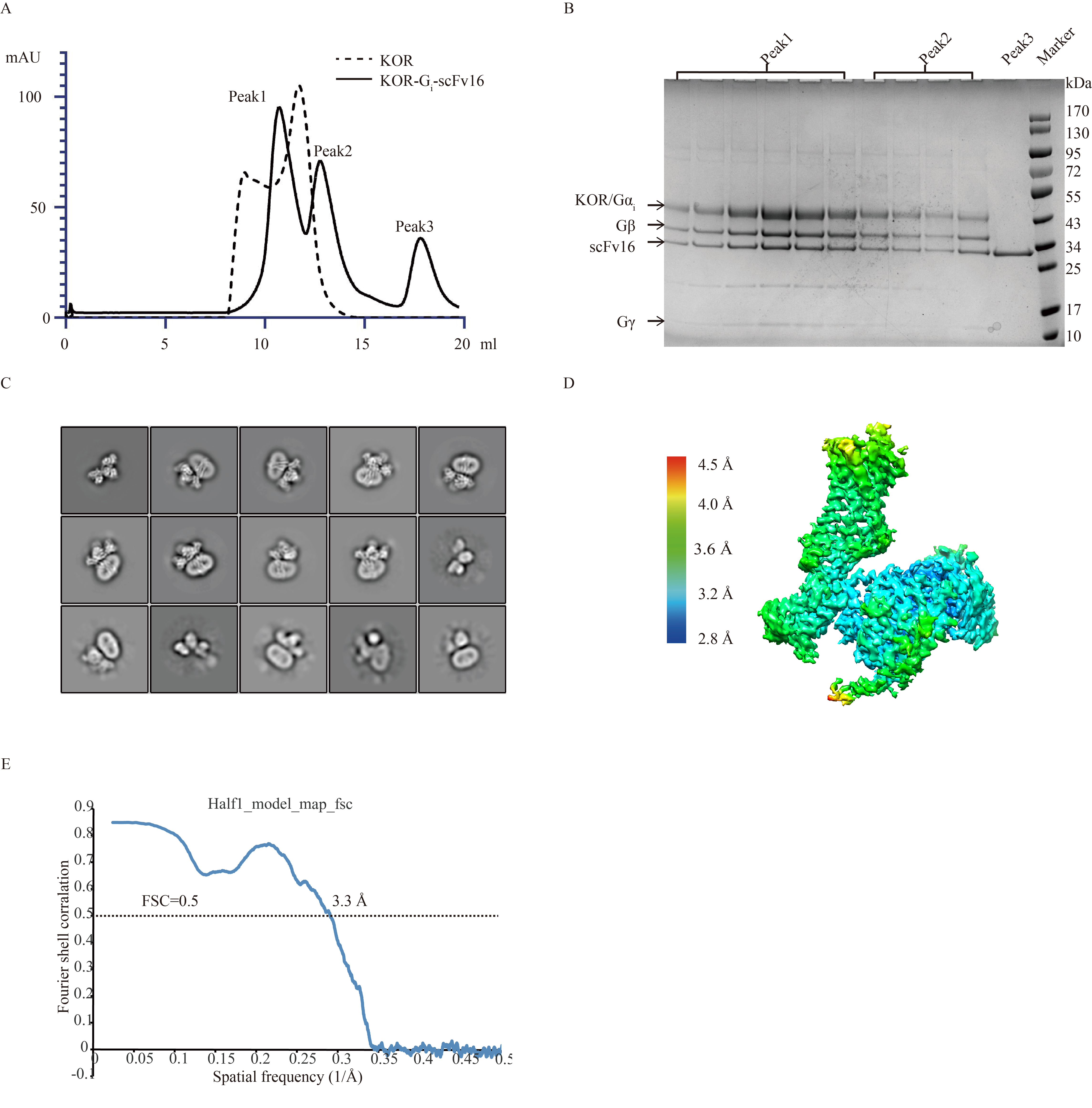


**Figure S1. Sample preparation and cryo-EM analysis of the dynorphin-bound KOR-G_i_-scFv16 complex.** (A) Size exclusion chromatography result for the preparation of KOR-G_i_-scFv16 complex. The dotted line and solid line represent KOR protein alone and KOR-G_i_-scFv16 complex, respectively. (B) Each peak fractions were subjected to SDS-PAGE analysis. Peak1, peak2 and peak3 represent the components of KOR-G_i_-scFv16, G_i_ and scFv16, respectively. KOR and Gα_i_ have the overlapping protein bands due to their similar molecular weight. (C) 2D class averages of the KOR-G_i_-scFv16 complex. (D) Final 3D density map colored according to the local resolution. (E) Half-1 model map FSC curves, showing the overall nominal resolution at 3.3 Å.


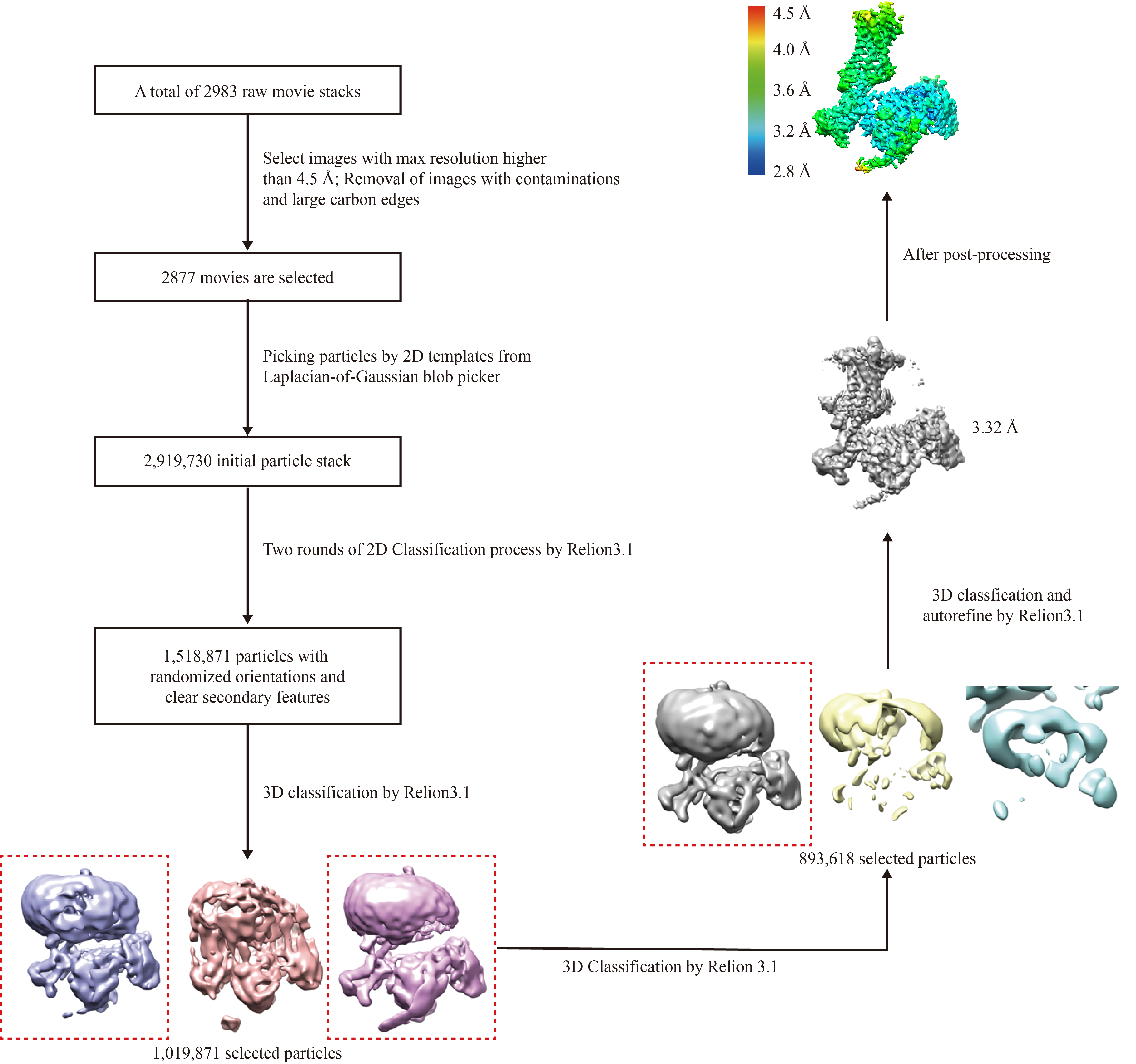


**Figure S2. Flow chart for the cryo-EM data processing and structure determination of the KOR–G_i_–scFv16 complex.** See Methods for details. The final reconstruction has an average resolution of 3.27 Å. All the images in this figure were created in UCSF Chimera.


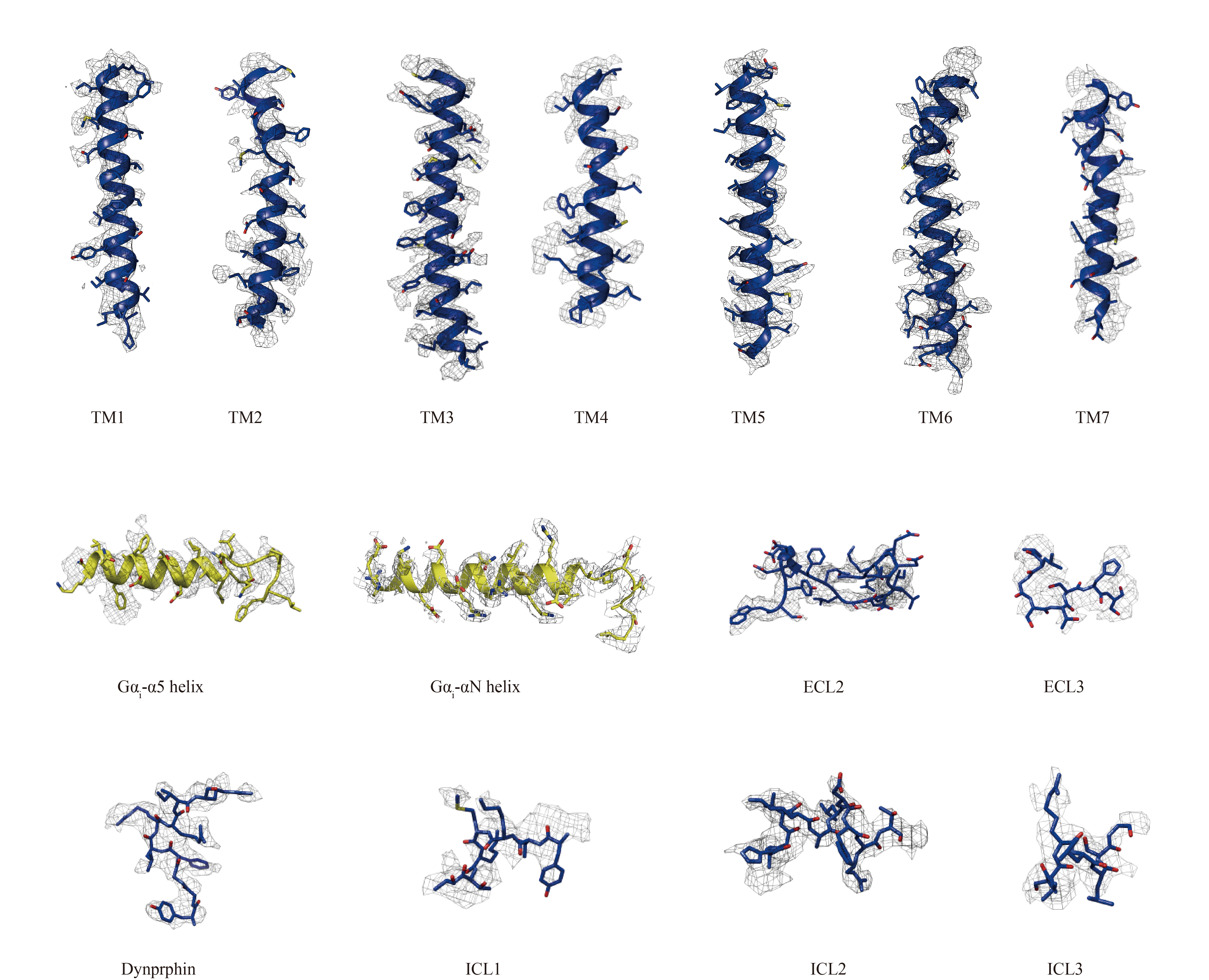


**Figure S3. Cryo-EM map quality assessment.** Cryo-EM density maps of seven helices, extracellular and intracellular loops for KOR (blue) and dynorphin (blue), α5 and αN helix of Gα_i_ (yellow).


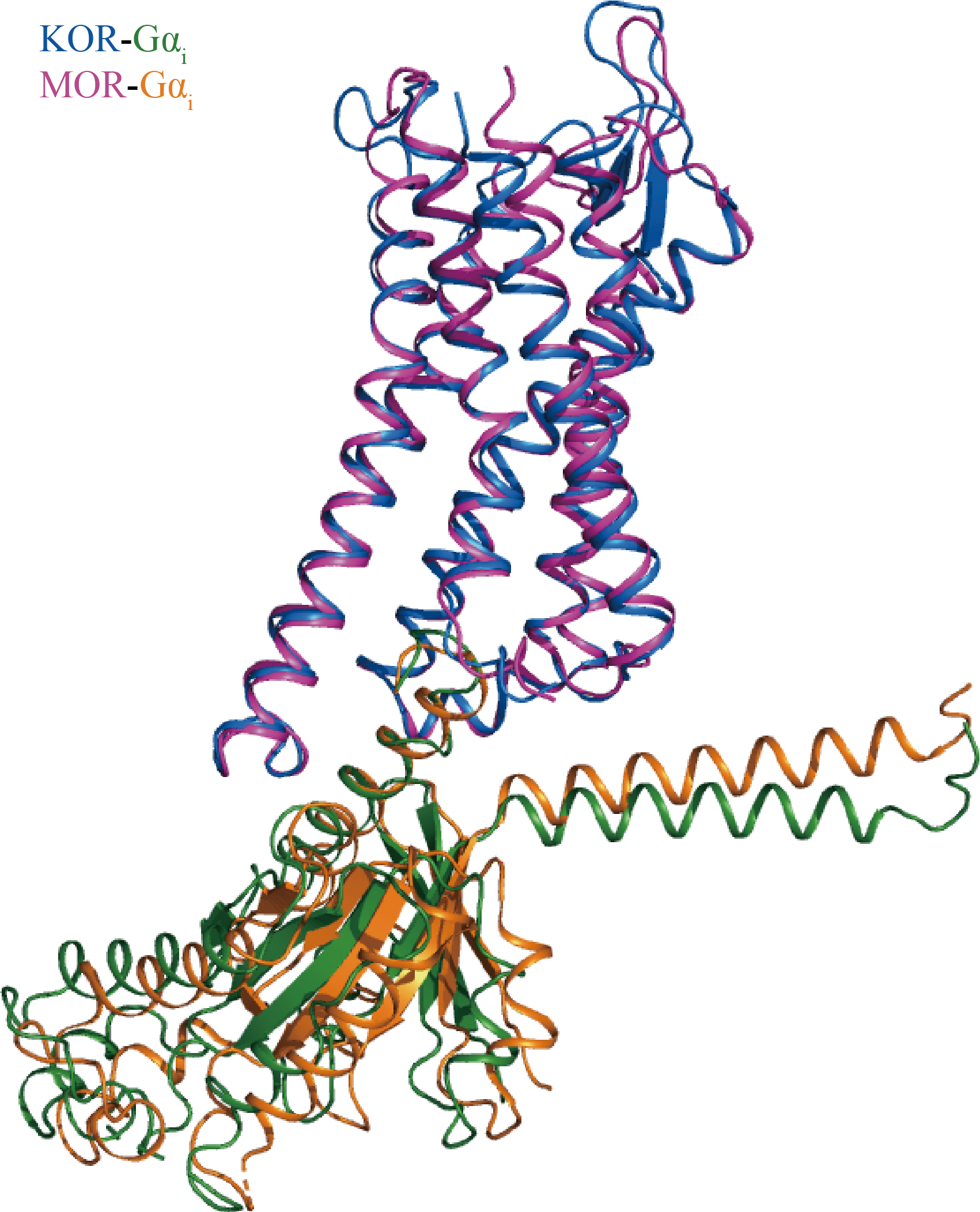


**Figure S4. Structural comparison between KOR-G_i_ (blue-green) and MOR-G_i_ (magenta-orange, PDB: 6DDE).**


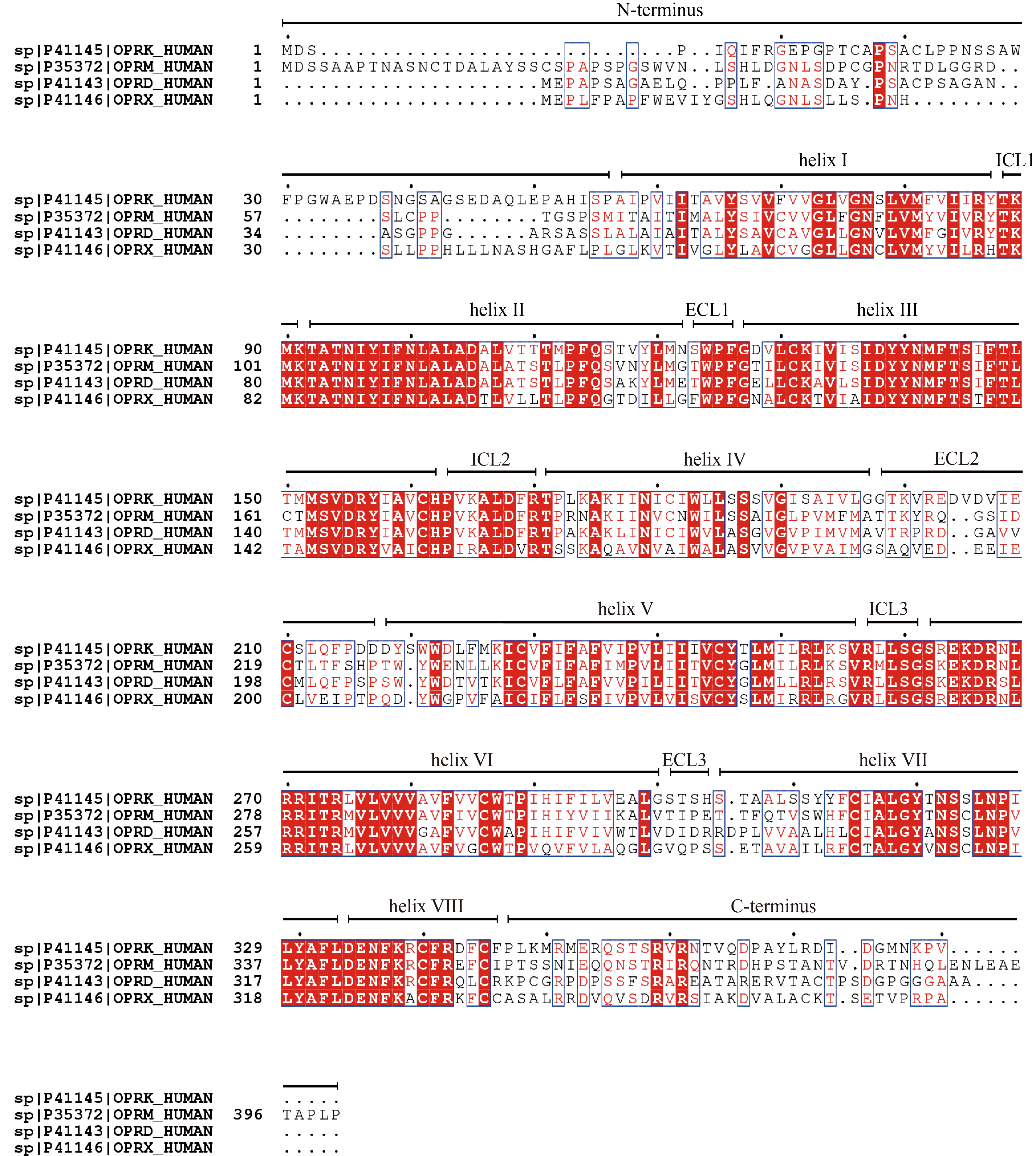


**Figure S5. Sequence alignment between KOR and three other opioid receptors.** OPRK, OPRM, OPRD and OPRX represent KOR, MOR, DOR and nociceptin receptor, respectively. Colors represent the similarity of residues: red background, identical; red text, similar. The alignment was generated using Clustal Omega (https://www.ebi.ac.uk/Tools/msa/Clustal Omega/) and the graphic was prepared on the ESPript 3.0 server (<http://espript.ibcp.fr/ESPript/cgi-bin/>ESPript. cgi).


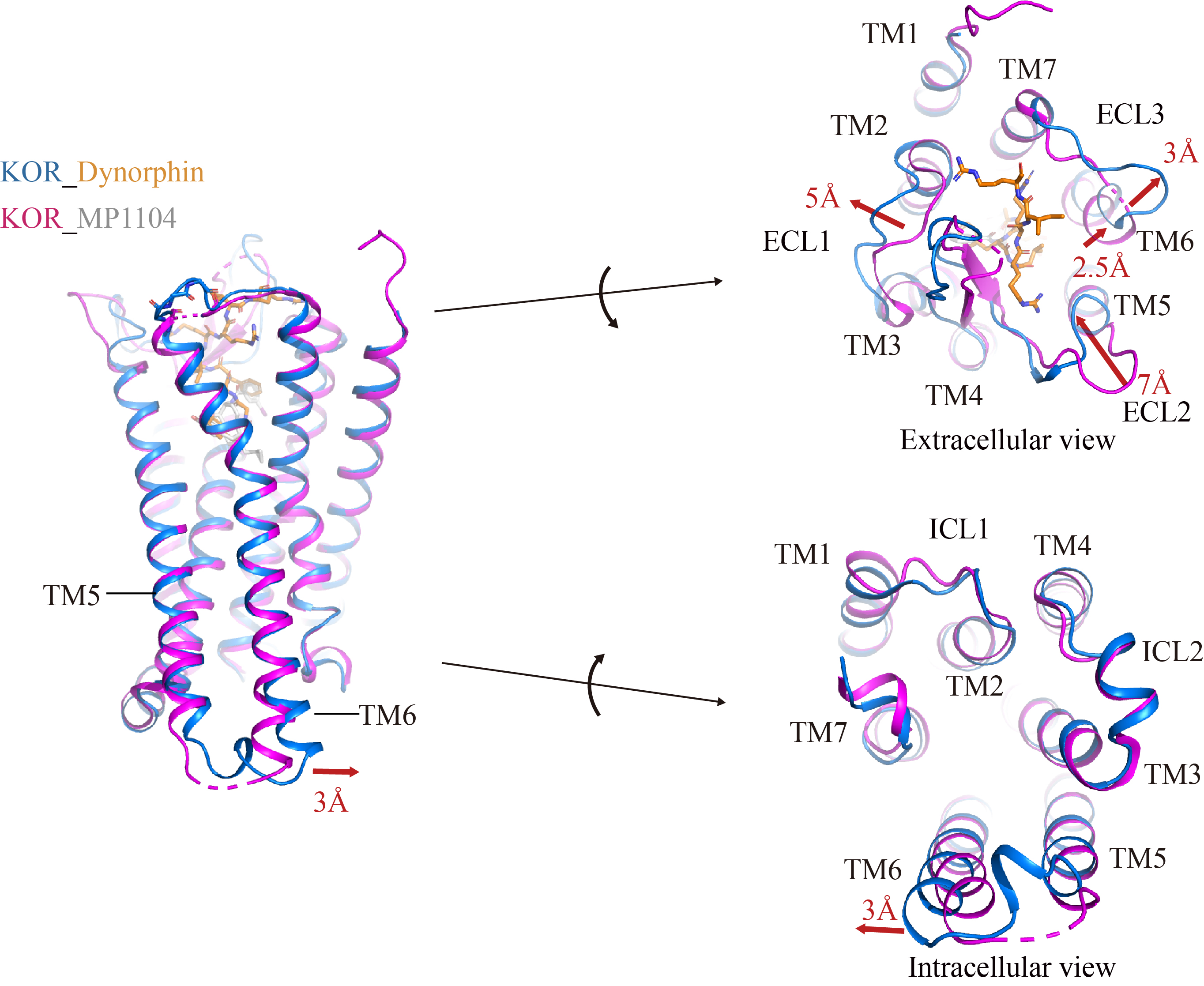


**Figure S6. Comparison of the helix movement between dynorphin-KOR (orange-blue) and MP1104-KOR (grey-magenta, PDB: 6B73) structures.**


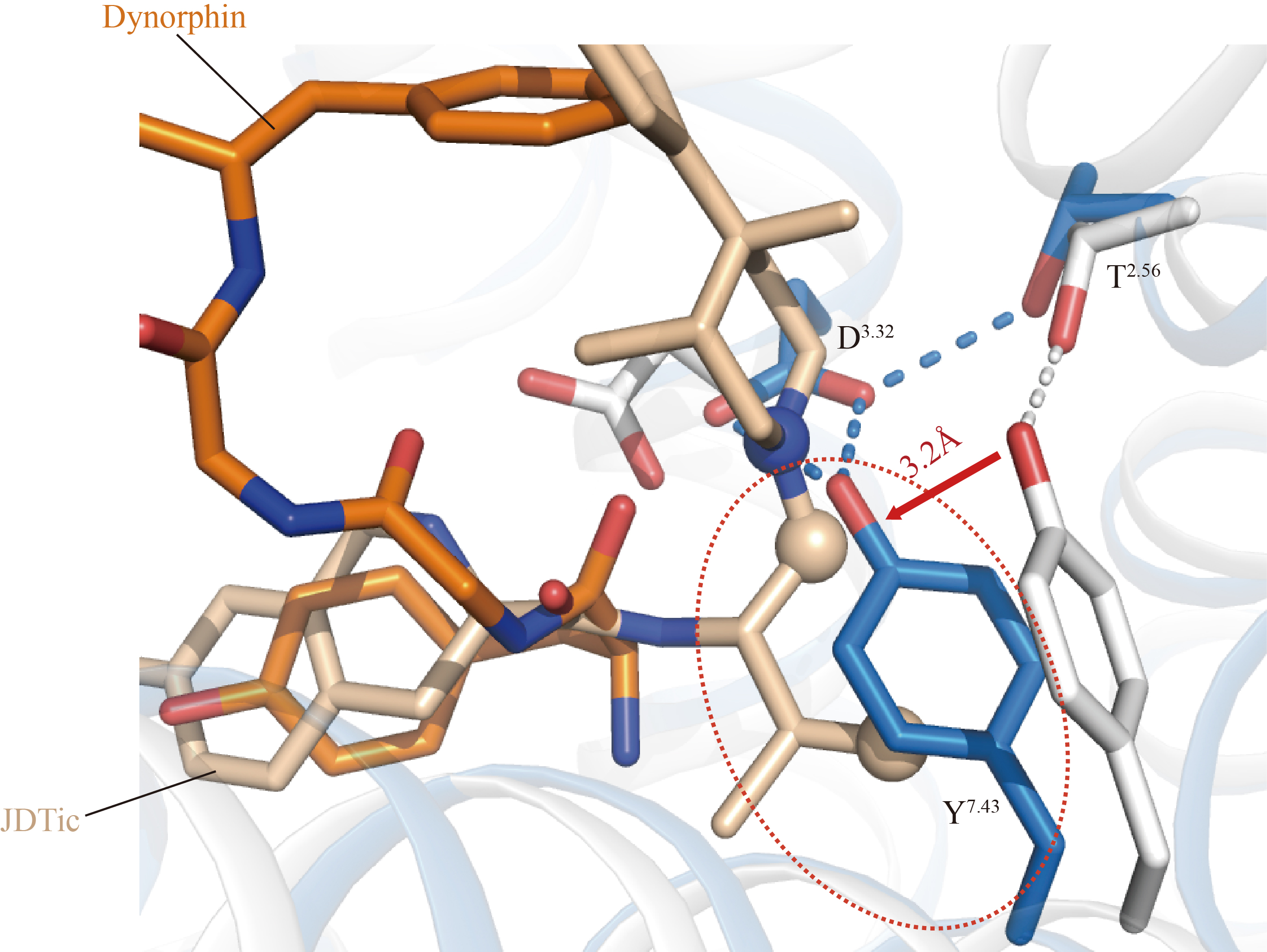


**Figure S7.** **Clash between JDTic and Y^7.43^.** Grey and blue sticks represent the corresponding residues in the JDTic-bound inactive-state KOR structure (PDB: 4DJH) and in the dynorphin-bound active-state KOR structure, respectively. Y^7.43^ forms steric clash (circled with dotted line) with JDTic (wheat) in the active-state KOR structure.

**Table S1. Cryo-EM data collection and refinement statistics**


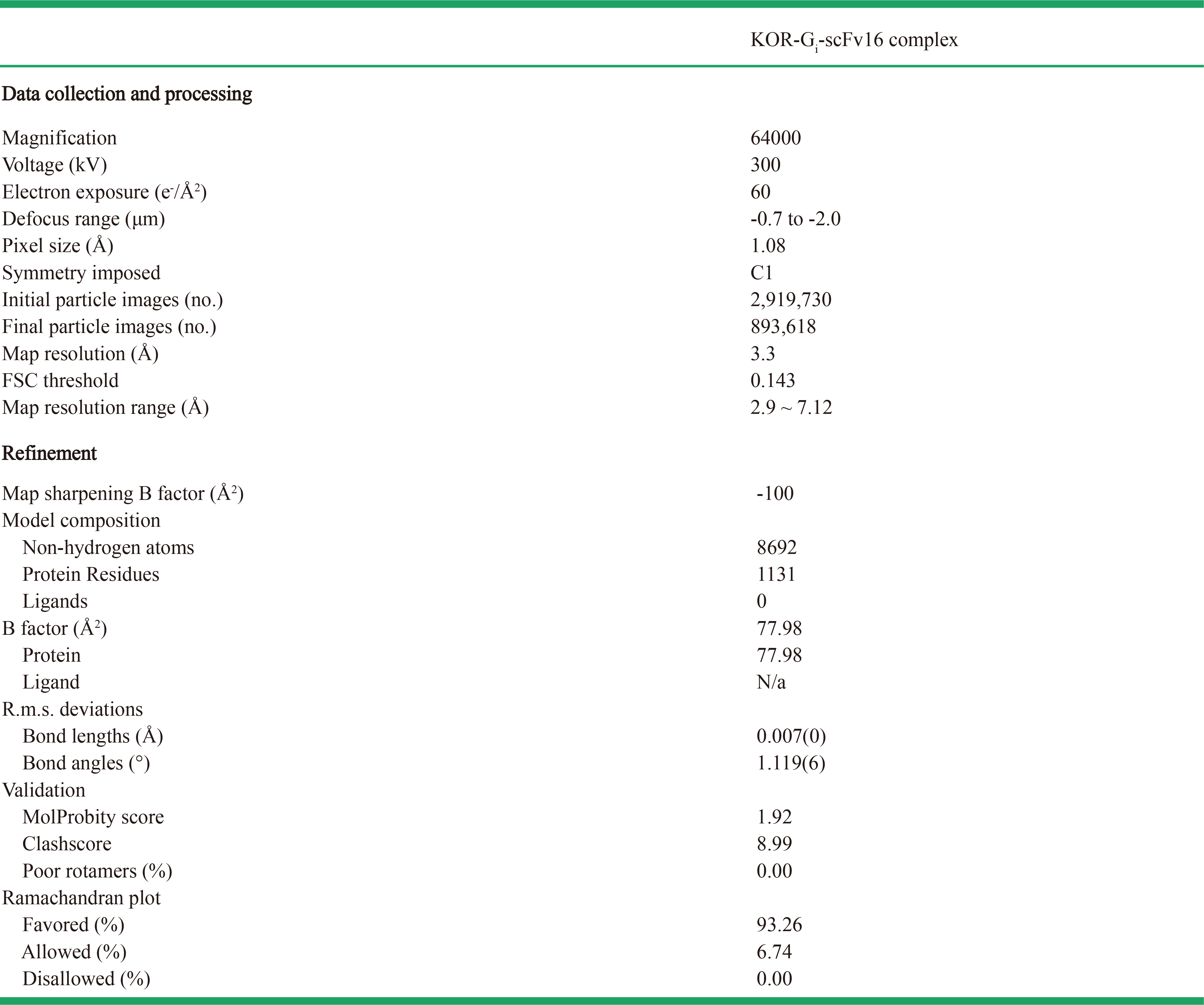


**Table S2. Summary of cAMP functional assay for the tested KOR mutants**


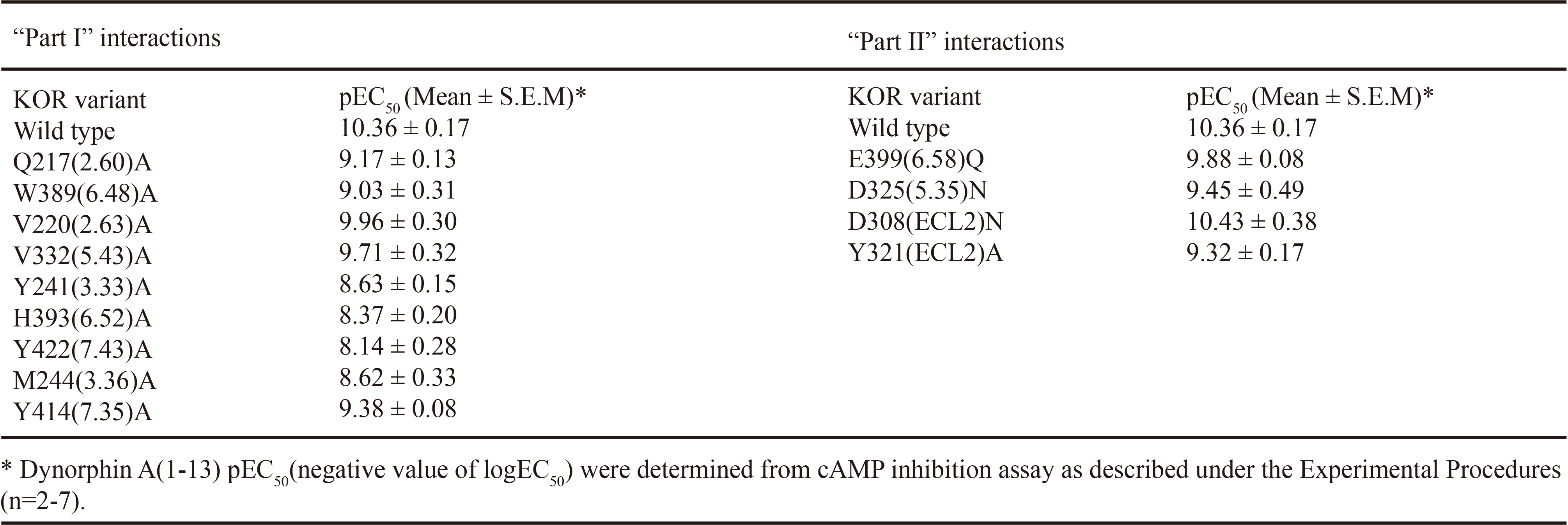

Supplement: pwac033_suppl_Supplementary_Material [file pwac033_suppl_supplementary_material.docx]
